# Electronic Health Records in Dentistry: Relevance, Challenges and Policy Directions

**DOI:** 10.1016/j.identj.2025.103964

**Published:** 2025-10-16

**Authors:** Falk Schwendicke, Sergio E. Uribe, Muhamad Walji, Walter Lam, Antonin Tichy

**Affiliations:** a Clinic for Conservative Dentistry, Periodontology and Digital Dentistry, LMU University Hospital, Munich, Germany; b Department of Conservative Dentistry and Oral Health, Riga Stradins University, Riga, Latvia; c Department of Clinical and Health Informatics, Department of Dental Informatics, UTHealth Houston; d Restorative Dental Sciences, Faculty of Dentistry, the University of Hong Kong, Hong Kong Special Administrative Region, China; e First Faculty of Medicine, Institute of Dental Medicine, Charles University, Prague, Czech Republic

**Keywords:** Data science, Electronic health records, Interoperability

## Abstract

Electronic health records (EHRs) have helped to transform modern healthcare by enabling structured, interoperable, and patient-centred information systems that connect providers, support research, and empower patients. Dentistry, however, has been slower to achieve full integration into national and international EHR frameworks, risking isolation from the broader health data ecosystem. This narrative review examines the global context of EHR development, the opportunities and risks for dentistry, and the policy directions that can guide successful integration. Drawing on international experience, national case studies, and the insights of both the FDI World Dental Federation’s Consensus Statement on Integrated EHRs and FDI’s Policy Statement on EHRs, the review highlights both the benefits and challenges of linking dental and medical records. Core oral and general health indicators that should be included in integrated systems are discussed, and relevant standards and interoperability frameworks presented. The paper concludes in a presentation of the FDI Policy Statement on EHRs, outlining practical recommendations, as well as a call to action to ensure that oral health is fully embedded within the digital health transformation. Comprehensive, secure, interoperable, patient-centred EHRs that integrate oral and systemic data on equal footing with medicine, built on common standards, auditable privacy and access controls, equitable patient and provider usability, and sustainable, routinely evaluated workflows, are essential to improve quality and safety of care, enable research and public-health monitoring, and support accountable, data-driven dentistry across settings.

## Introduction

The digital transformation of healthcare has been one of the most profound shifts in medical practice in recent decades. Electronic Health Records (EHRs) have evolved from isolated, localised systems into complex, networked platforms capable of capturing a patient’s journey across different levels and streams of care. For medicine, this has become the norm in some regions and an aspiration in others. In dentistry, however, progress has been uneven, and the discipline’s data often remains siloed in a mix of paper and electronic dental records that are not interoperable with wider health systems.

This separation increasingly contradicts the scientific consensus that oral health is integral to general health. Periodontal disease is linked with diabetes; oral manifestations can be early signs of systemic conditions, while systemic conditions and treatments, ranging from cardiovascular disease to cancer therapy, directly affect oral health and influence dental treatment planning. In the absence of integrated records, these connections are harder to leverage in day-to-day care, research, and health system planning. In a U.S. cohort with nonintegrated records, 15.1% of patients with diabetes and 29.0% with hypertension failed to disclose these conditions to their dentists; gaps that integrated medical–dental EHRs could help prevent.^1^

The FDI World Dental Federation’s Consensus Statement on Integrated EHRs^2^ has made clear that dentistry’s separation from mainstream EHRs is neither inevitable nor acceptable. Instead, it frames integrated EHRs as a component of holistic, person-centred care, aligned with global health strategies such as the WHO’s Global Strategy on Oral Health 2023-2030 and the Bangkok Declaration of 2024. This review builds on that perspective, setting out the opportunities, risks, and pathways for dentistry to join the digital health mainstream.

## Context and concept

EHRs should be more than just digital versions of paper files. At their most advanced level, they are structured, interoperable systems that allow health information to flow seamlessly between providers, institutions, and jurisdictions. In dentistry, digital records are becoming more widespread at the practice level, but most are electronic dental records, ie, systems optimised for recording odontograms, radiographs, and procedure codes, but not designed for integration with medical data or for further use beyond clinical care.

An integrated EHR system goes further; it combines dental and medical records in a single, secure platform, enabling shared access for all authorised members of a patient’s care team. It ensures syntactic interoperability (see below) by supporting standardised data formats and exchange protocols so that information can move seamlessly across systems. At the same time, it enables semantic interoperability using standardised coding systems for diagnoses and procedures, ensuring that the meaning of oral health data is preserved and correctly interpreted in different contexts. In doing so, such systems facilitate both primary use (direct patient care) and secondary use (research, policy, innovation). The FDI’s Consensus Statement emphasises that such integration is not a technical luxury but a necessity, ensuring that oral health data both informs and is informed by medical decision-making.^2^

Despite the clear rationale, the global picture remains mixed. While certain countries have achieved near-universal EHR adoption in medicine, dental integration lags behind. In 2012, only 52% of U.S. dental practices used any form of electronic record system for patient care,^3^ and even fewer were linked to medical records. In Europe, a 2017 survey found many countries still in the planning stages for national EHRs, with limited attention to dental data.^4^

## Opportunities

The potential benefits of integrated EHRs for dentistry are extensive. Clinically, they allow dentists to access a richer health history, from laboratory results to medication records, that can inform safe and effective treatment. A dentist aware of a patient’s anticoagulant use, recent cardiac events, or immunosuppressive therapy can plan surgical procedures with reduced risk. Similarly, medical colleagues can benefit from timely dental data, such as evidence of periodontal inflammation that may affect diabetes control or clearance for surgical procedures.

Integrated EHRs also enable safer prescribing through automatic alerts about drug interactions, duplicate therapies, potential allergies, and other adverse effects. For example, an alert about a patient’s bisphosphonate use can inform the management of invasive dental procedures to prevent osteonecrosis of the jaw.

Beyond direct care, integrated EHRs enhance research capacity. Large, standardised datasets allow epidemiologists to explore oral–systemic health relationships at scale and give health services researchers the data needed to evaluate interventions.^5^ They also underpin AI-driven tools that can assist in diagnosis, treatment planning, and risk prediction. One such prominent example where EHRs flow into relevant oral health research is BigMouth,^6^^,^^7^ a large-scale, centralized data repository that pools EHR data from participating dental schools and academic institutions. It enables researchers to access a rich, standardized dataset covering a wide spectrum of clinical, demographic, diagnostic, and procedural information related to oral health, disease, and treatment. By harmonizing data from multiple sources, BigMouth supports multi-institutional studies, facilitates epidemiological analyses, and accelerates evidence generation for clinical decision-making and policy development. Its secure infrastructure ensures patient privacy while fostering collaboration among dental researchers, ultimately contributing to a deeper understanding of oral health trends, disparities, and outcomes.

EHRs further have the potential to transform public oral health by turning routine clinical data into a powerful resource for surveillance and policymaking. When aggregated across providers, EHR data enable near real-time monitoring of disease prevalence, treatment patterns, and emerging concerns, offering greater speed and granularity than traditional surveys, which increasingly struggle to attain representative population samples. Estonia demonstrates successful national integration since 2008, with mandatory provider participation achieving nationwide interoperability through secure cross-border data sharing (https://ec.europa.eu/digital-building-blocks/sites/spaces/DIGITAL/blog/2019/07/26/533365863/Estonian+Central+Health+Information+System+and+Patient+Portal).

Because EHRs often reflect demographic and socioeconomic details, they can reveal disparities in access, disease burden, and outcomes, guiding targeted prevention and outreach efforts for underserved communities. Large-scale datasets also allow researchers to evaluate the effectiveness of public health policies, track the impact of interventions such as insurance coverage expansions, and study disease progression over time to build predictive models for prevention and care. Finally, patient engagement is strengthened through portals and personal health records that allow individuals to access their information, contribute to shared decision-making, and manage their health more proactively.

## Risks and challenges

The pathway to integration is not without obstacles. Technical barriers include incompatible data formats, lack of standardisation in dental coding, and legacy systems without interoperability capabilities (see below). International disparities add another layer of complexity. While, for example, the European Health Data Space (EU EHDS) offers a coordinated framework for the European Union, many low- and middle-income countries lack national EHR strategies, let alone dental integration. Without deliberate action, the digital divide could widen, leaving some dental communities behind.

Legal and ethical issues are also significant. Privacy regulations vary between jurisdictions, creating uncertainty about how health records can be lawfully collected, stored, and shared. Moreover, the inclusion of dental data may increase the risk of re-identification, as certain dental features are highly distinctive, which is why dental records are commonly used in forensics. This raises important questions about who should be entitled to access complete patient records and how secondary uses of such data should be governed. Patients may be reluctant to consent to data sharing if the risks are not clearly communicated, and clinicians may be hesitant to participate if the legal frameworks remain ambiguous. Security breaches further intensify these concerns, potentially undermining public trust and jeopardising the widespread adoption of EHR systems.^8^

Data integrity faces additional threats from governmental manipulation, where authorities may alter health datasets for political purposes,^9^ compromising research validity and public trust. Blockchain technology offers protection by creating permanent, unalterable records through cryptographic signatures, with each data entry receiving a unique digital fingerprint that changes if modified, while multiple computers verify these records, making unauthorized changes detectable.

Practical challenges include the cost of upgrading systems and training staff, workflow disruptions during implementation, and resistance from stakeholders who see limited short-term benefit. Smaller practices may lack the resources to invest in integrated platforms without external support. Burnout has also been associated with EHR use.^10^

Data quality presents another fundamental challenge, as current EHRs often serve primarily billing and administrative functions rather than direct clinical care or secondary research. An analysis of the BigMouth database with over 500,000 patients found high completeness for demographic variables (97.6%-99.9%) but dramatically lower completeness for clinical variables: tobacco use (32.1%), alcohol use (45.3%), and pain rating (1.5%-2.0%).^11^ This creates systematic gaps in clinical information needed for comprehensive patient care, the development of safe predictive models and research.

Integrated records may also introduce socio-technical challenges. In an environment where EHR alerts are often overridden or ignored, how should prompts for periodontal co-management in patients with diabetes be delivered without adding to alert fatigue? Likewise, if a patient seen in a dental clinic is overdue for a colonoscopy, what responsibility does the dentist have to initiate or facilitate that preventive screening, and through what closed-loop process? Realizing the full benefit of integrated medical–dental EHRs will require further study to identify which workflows, alerts, and referral pathways translate into measurable gains in safety, equity, and patient outcomes.

## Health indicators

One main question is what to record. As outlined above, there is great variability across countries and jurisdictions. FDI’s Consensus Statement^2^ recommends prioritising a focused set of oral and general health indicators to ensure integration is clinically meaningful without overwhelming systems with excessive data (Table 1).

**Table 1:** Consented health indicators to be collected in EHRs as suggested by FDI.

Category	Recommended indicators
Oral health	• Caries and endodontic health (dental decay, root-related conditions)
	• Periodontal disease indicators (eg, gum health, inflammation)
	• Oral cancer screening/examination results
	• General oral health status measures (overall assessment, promotional needs)
	• Information on medical devices and implants (eg, dental prostheses, implants)
	• Prescription data relevant to oral health (eg, medications prescribed by dentists)
	• Allergy information and medical history findings (eg, material or drug allergies relevant to dental care)
	• Radiographs and diagnostic imaging (dental radiographs, scans)
	• Salivary health markers (flow rate, pH levels, buffer capacity)
	• Masticatory function measures (bite force, chewing efficiency)
	• Preventive care engagement (fluoride exposure, oral hygiene practices, dietary patterns)
	• Social determinants (food security status, oral health literacy levels, access barriers)
	• Functional outcomes (speech clarity, swallowing function, aesthetic satisfaction)
General health	• Patient identification and demographic details (for accurate linking across systems)
	• Medical history and diagnoses (overall health conditions impacting dental care)
	• Prescription and medication data (overall medication profile, drug interactions)
	• Allergies and medical alerts (across general and dental care contexts)
	• Radiographs and diagnostic images (medical imaging relevant to overall patient care)
	• Functional and risk indicators (eg, risk factors or functional limitations that may affect dental care)

Current oral health surveillance focuses on the absence of disease rather than the presence of health. EHRs provides an opportunity to track health through positive indicators that capture functional capacity and biological resilience. Dental EHRs may also want to record salivary biomarkers, masticatory efficiency and functional outcomes including chewing ability and speech, as well as social determinants including food security or oral health literacy.

## Standards and interoperability

A critical enabler of integrated electronic health records is the use of consistent, internationally recognised terminologies, ontologies, and data exchange formats.^12^ Without them, even the most advanced technical platforms cannot communicate effectively, leading to fragmented records and duplication of effort. The following sections present a range of relevant frameworks for syntactic and semantic interoperability.

Data exchange formats and messaging standards define the syntax and structure of how information is packaged and transmitted between systems, ensuring that records can be shared consistently regardless of vendor or platform. HL7 v2 remains widely used for administrative messaging; HL7 CDA structures clinical documents; and the newer HL7 FHIR enables modern, granular, web-based data exchange. FHIR is central to EU EHDS planning and U.S. American Dental Association (ADA) pilot projects. Imaging interoperability relies on DICOM, while IHE profiles define consistent workflows for integration.

Semantic interoperability depends on the consistent use of terminologies and ontologies. The International Classification of Diseases (ICD), now transitioning from ICD-10 to ICD-11 in some countries, is widely used but has limited granularity for dental diagnoses. SNOMED Clinical Terms (SNOMED CT), in contrast, offers a detailed, concept-based structure covering dental and medical conditions. SNODENT, created by the ADA, is a dental-focused subset aligned to SNOMED CT for U.S. practice, while Current Dental Terminology (CDT) supports billing and insurance processes but lacks semantic interoperability. Mapping between ICD, SNOMED CT, SNODENT, and CDT is challenging, often leading to loss of clinical detail and inconsistencies in analytics. Licensing restrictions can further hinder adoption.

Notably, partial or inconsistent implementation of these terminologies and standards by software vendors, lack of awareness among dental professionals, and resource constraints limit the real-world impact of these standards. Yet their adoption is fundamental for cross-disciplinary, internationally compatible dental EHRs.

## Implementation strategies

Achieving integration requires coordinated action. Stakeholder engagement is essential from the earliest stages, involving not only dentists and physicians but also IT professionals, regulators, patients, and industry partners. Education and training are critical, with digital health competencies embedded in dental curricula and offered through continuing professional development. Pilot programmes can test integrated systems in real-world settings, identify challenges, and refine workflows before wider rollout. Monitoring and evaluation should be continuous, measuring not only technical performance but also clinical outcomes and patient satisfaction. Ethical frameworks must underpin all efforts, ensuring robust security, transparent consent processes, and equitable access. Financing strategies may include government incentives, insurance-linked models, public–private partnerships, value-based care frameworks, and research grants. Without adequate funding, especially for smaller practices, integration efforts are unlikely to succeed; the burden of implementing EHRs cannot be shouldered by clinicians, whose primary task is clinical care. Overall, the acknowledgement of both opportunities and challenges (Table 2) remains crucial to achieve full-scale implementation of EHRs.

**Table 2:** Opportunities and challenges for EHRs in dentistry.

Opportunities	Challenges
Improved care coordination between dental and medical providers	Incompatible data formats and lack of dental coding standards
Safer medication management and reduced errors	High cost of upgrading systems and infrastructure
Enhanced research capacity and public health surveillance	Stakeholder resistance and workflow disruption
Greater patient engagement through access to complete records	Variability in privacy laws and regulatory frameworks
Support for AI-driven diagnostics and predictive analytics	Digital divide between high- and low-resource settings
Standardised oral health indicators for global comparison	Limited dental representation in national policy-making

## FDI policy statement on EHRs

The FDI’s Policy Statement on EHRs (REF TO BE INSERTED) is a formal articulation of the dental profession’s position on how EHR systems should be developed, implemented, and maintained to serve both clinical and public health needs. Emerging from global consultation, and recognising the accelerating digital transformation of healthcare, the statement addresses the urgent need for dentistry to be integrated into national and international health information infrastructures.

At its core, the policy statement calls for EHR systems to be comprehensive, secure, interoperable, and patient-centred. It stresses that dental data must not be an afterthought in health record design but should be incorporated from the outset, with parity to medical data in terms of access, quality, and protection. This principle reflects the mounting evidence that oral health and systemic health are closely interconnected, and that integrated data can support better prevention, diagnosis, and management of disease. The policy statement also recognizes the international diversity in scope and implementation of EHRs in oral health.

FDI’s vision for EHRs rests on 3 main pillars:

• *Building trust*: Secure handling of data, compliance with relevant laws, and ensuring confidentiality, accessibility, and traceability are essential to maintain confidence among patients, providers, and policymakers.
• *Improving care*: Systems should enhance quality, safety, and accessibility of dental services, while enabling research through robust consent frameworks and ensuring compatibility with established standards.
• *Ensuring sustainability*: EHR systems should be practical, cost-effective, user-friendly, and designed for long-term adoption.

FDI calls on EHR designers, users, policymakers, and the research community to:

• *Protect privacy and security*: Confidential information must be securely stored and transmitted, with clear, enforceable access rights and audit capabilities to track changes.
• *Promote equity and accessibility*: EHRs should advance universal health coverage goals and avoid worsening disparities. Patients must be able to access their own records without significant obstacles, and dental professionals should be trained to use EHRs responsibly while respecting local privacy laws.
• *Maximize value*: Digital records should underpin a more data-informed model of oral health care and research, enabling safer, more personalized treatment. Public health programs and disease monitoring should be strengthened through the use of de-identified, transparently shared data. When data are used for commercial purposes, the individuals providing it should receive fair benefits.
• *Enable data-driven health systems*: Adoption of a common data model and internationally recognized interoperability standards, such as FHIR and DICOM, is essential for connecting oral health data to wider health systems.
• *Evaluate impact*: Development should be guided by multidisciplinary teams to ensure EHRs are efficient, intuitive, and fit for purpose. Costs must be factored in, and oral health information should be seamlessly integrated with medical records to promote holistic, person-centered care.
• *Conduct ongoing review*: Systems should be regularly assessed for their effects on patient record management, administrative processes, data integrity, and clinical workflows.

By situating dentistry within the broader digital health policy landscape, the FDI Policy Statement functions as both a benchmark and a call to action. It is adaptable to national contexts, serving as a framework for enhancement in high-resource settings and as a roadmap for incremental progress in resource-limited contexts.

## Conclusion

The integration of dentistry into mainstream EHR frameworks is both a challenge and an opportunity. The evidence and experience outlined in this review show that integration is achievable, beneficial, and increasingly necessary. Dentists should advocate for inclusion in national and regional EHR strategies, adopt standardised indicators, and participate in interoperability testing. Policymakers should legislate for dental integration and provide the resources needed to make it a reality. Researchers should prioritise studies that evaluate integration efforts and their impact on care and health outcomes. Industry should work collaboratively with end-users to develop systems that are secure, interoperable, and fit for purpose.

EHRs have the potential to transform dentistry, connecting it fully to the wider health system and enabling more holistic, efficient, and patient-centred care. The global momentum towards integrated health data systems provides an opportunity that dentistry cannot afford to miss. By following the pathways outlined in this review, and guided by the recommendations of FDI, the dental profession can ensure that oral health is embedded at the heart of the digital health transformation.

## Conflict of interest

The authors declare no conflicts of interest.
